# Association of IL-1β rs16944 polymorphisms and the subsequent level of IL-1β in type 2 diabetes mellitus: Case-control study

**DOI:** 10.1097/MD.0000000000045029

**Published:** 2025-10-24

**Authors:** Haider Mohammad Khdaer, Muhammed A.H. Aldabagh, Zeyad T. Abdulrazzaq, Galal A. Altai, Ali Abdullah Issa, Majid S. Jabir, Mazin A.A. Najm, Thikra F. Hasan, Sabrean F. Jawad, Hayder Adnan Fawzi, Samer Tareq Jasim, Ayman A. Swelum

**Affiliations:** a Department of Medicine, Al-Shaheed Mohammad Baqir Al-Hakim Hospital, Baghdad, Iraq; b Department of Research Unit, College of Medicine, Al-Nahrain University, Baghdad, Iraq; c Department of Physiology, College of Dentistry, Al-Mustafa University, Baghdad, Iraq; d Department of Internal Medicine, College of Medicine, Al-Nahrain University, Baghdad, Iraq; e Department of Applied Science, University of Technology, Baghdad, Iraq; f Department of Pharmaceutical chemistry, Mazaya University College, Dhi-Qar, Iraq; g Department of Immunology, Uruk University, Baghdad, Iraq; h Department of Biochemistry, College of Pharmacy, Al-Mustaqbal University, Hillah, Babylon, Iraq; i Department of Clinical Pharmacy, College of Pharmacy, Al-Mustafa University, Baghdad, Iraq; j Department of Pharmacetical Cheistry, Pharmacy College, Imam Ja’afar Al-Sadiq University, Baghdad, Iraq; k Department of Animal Production, King Saud University, Riyadh, Saudi Arabia.

**Keywords:** insulin resistance, interleukin-1β, polymorphisms, rs16944, type 2 diabetes mellitus

## Abstract

Strong relationships exist between immune factors and hyperglycemia, particularly proinflammatory factors such as interleukin (IL)-1β protein levels and genetics, which significantly correlate with the development of type 2 diabetes mellitus (T2DM). Exposure of pancreatic cells to IL-1β for an extended period enhances β-cell apoptosis, thereby affecting glucose metabolism. The study objective is to assess the functional role of IL-1β protein levels and the IL-1β rs16944 gene polymorphism in T2DM. A case-control study was conducted, involving 86 participants with T2DM and 74 controls. Blood samples were collected from the participants to assess their IL-β1 levels, fasting blood sugar levels, and fasting insulin levels. IL-1β (rs16944) was analyzed using conventional polymerase chain reaction. IL-1β rs16944 variations in the control group showed 32 (43%) of AA, 27 (36%) of AG, and 15 (20%) of GG, while T2DM patients showed 30 (35%) of AA, 34 (40%) of AG and 22 (26%) of GG variation. The logistic regression analysis showed a non-significant association between each variation and T2DM. The genome variation in patients with insulin resistance (IR) and T2DM was 8 (33%) AA, 6 (25%) AG, and 10 (42%) GG. The logistic regression test showed a non-significant association between each variation and patients with IR and T2DM. In conclusion, IL-1β levels and genetic variations of the IL-1β rs16944 gene do not affect blood sugar metabolism and I nsulin resistance in Iraqi diabetic patients.

## 1. Introduction

Type 2 diabetes mellitus (T2DM) is a significant global disease characterized by elevated blood glucose levels.^[1,2]^ According to the International Diabetes Federation, the global prevalence of T2DM in 2020 was roughly 463 million patients^[3]^; according to a 2018 study from the World Health Organization, over 1.4 million individuals in Iraq are diagnosed with diabetes.^[4]^ Several factors may increase the risk of developing T2DM, including obesity, hypertension, lifestyle, age, nutrition, physical activity, and genetic predisposition.^[5–9]^ Accumulated evidence reported strong relationships between immune factors and hyperglycemia.^[10–13]^

Interlukin (IL)-1β is mainly produced by dendritic cells, blood monocytes, and tissue macrophages.^[14,15]^ The chronic elevation of IL-1β in pancreatic cells causes β-cell apoptosis, impairs insulin secretion, and causes a gradual loss of β-cell mass that contributes to T2DM.^[16–18]^ IL-1β is a major proinflammatory cytokine, playing a wide role in autoinflammatory diseases, and acts as a key promoter of systemic and tissue inflammation in T2DM.^[19,20]^ There is some evidence indicating that elevated glucose levels may cause the Nod-like receptor family pyrin domain-containing 3 inflammasome to activate in islet β-cells, leading to the secretion of IL-1β.^[21,22]^ When the Nod-like receptor family pyrin domain-containing 3 inflammasome is activated, it can induce programmed cell death (apoptosis) in β-cells, which further reduces the overall β-cell mass.^[21,23]^

The IL-1β (rs16944) variant is a single-nucleotide polymorphism (SNP) in the IL-1β gene, which encodes a pro-inflammatory cytokine involved in various immune and inflammatory responses. This SNP is located in the promoter region of the IL-1β gene, specifically at position -511 of the gene’s transcription start site.^[16]^ The IL-1β (rs16944) variant has been investigated in relation to T2DM; IL-1β is a proinflammatory cytokine, and inflammation plays a role in the pathogenesis of T2DM.^[24]^ The IL-1β gene variant rs16944 is located in the promoter region, and it can affect the production and activity of IL-1β.^[25]^ The activity associated with rs16944 can affect the gene’s expression and, consequently, the levels of IL-1β produced. Variants in this SNP have been linked to differences in inflammation and susceptibility to various diseases. For example, certain alleles at this SNP can influence an individual’s risk for conditions such as rheumatoid arthritis, inflammatory bowel disease, and cardiovascular diseases.^[26]^

Research has shown that certain alleles of the IL-1β (rs16944) SNP may be associated with increased levels of IL-1β and altered inflammatory responses. Elevated inflammation has been linked to insulin resistance (IR) and β-cell dysfunction, key factors in developing T2DM.^[27]^ However, the relationship between IL-1β (rs16944) and T2DM is complex and influenced by multiple genetic and environmental factors. While some studies have found associations between this SNP and an increased risk of developing T2DM, others have found weaker or no associations.^[28,29]^ Overall, IL-1β (rs16944) is one of many genetic factors that may contribute to diabetes risk, and its impact needs to be considered in the broader context of genetic predisposition and lifestyle factors. This study aims to investigate the functional role of IL-1β protein levels and the IL-1β (rs16944) gene polymorphism in patients with diabetes mellitus.

## 2. Material and methods

### 2.1. Study design and settings

A case-control research investigation was conducted on 160 Iraqi individuals aged between 30 and 70. Among them, 86 were diagnosed with T2DM by consultant internists, while the remaining 74 were healthy volunteers. The study took place at Al-Emamain Al-Kadhemain Medical City in Baghdad from December 2022 to March 2023. Participants were categorized based on their homeostatic model assessment for IR into 2 groups: IR (24 participants) and insulin sensitivity (62 individuals).

### 2.2. Inclusion criteria

The patient was diagnosed with T2DM.

### 2.3. Exclusion criteria

Patients presenting with acute inflammation and their relatives will be excluded from both the case and control groups.

### 2.4. Laboratory analysis

Each individual obtained 5 mL of venous blood, with 2.5 mL being transferred to an EDTA tube for biomarker measurement. The blood specimens were gathered during their regular hospital visit between 09:00 AM and 12:00 PM. The sera were separated from the remaining 2.5 mL of blood and preserved for biochemical and immunological tests.

A biochemical assay (Human, Germany) measured fasting blood sugar (FBS), while enzyme-linked immunosorbent assays (Sunlong, China) measured fasting insulin and IL-1β levels according to the manufacturer’s instructions.

### 2.5. DNA extraction

Genomic DNA was collected from blood samples (Frozen Blood) by the (gSYAN Geneaid kit, New Taipei City, Taiwan) extraction kit, and carried out following the company instructions as follows:

1. The sterile 1.5 mL Microcentrifuge tube was filled with 200 µL of frozen blood, then 30 µL of proteinase K was added and vortexed. Five minutes of incubation at 60°C followed.
2. Following this process, 200 µL GSB lysis buffer was inserted into each tube and vigorously mixed by a vortex. Each tube was incubated for ten minutes at 70°C and, during the incubation period, inverted every 3 minutes.
3. The lysate was loaded with 200 µL of absolute ethanol and then vigorously shaken.
4. The DNA filter column was installed in a 2-mL tube, and all the mixture (including any residue) was transferred to the column. It was centrifuged for 5 minutes at 10,000 rpm. The tube containing 2 mL of the flow-through was removed, and the column was inserted into a new tube containing 2 mL.
5. A 400 µL W1 buffer was also inserted into the DNA filter column and centrifuged for thirty seconds at 10,000 rpm. The flow-through was removed, and the column was inserted into the 2 mL tube.
6. 600 µL wash buffer (ethanol) was inserted in each column. The column was centrifuged for thirty seconds at 10,000 rpm. The flow-through was removed, and the column was inserted into the 2 mL tube.
7. All the tubes were centrifuged for 3 minutes at 10,000 rpm to dry the column and the matrix.
8. The dry DNA filter was moved into the clean 2 mL microcentrifuge tube, and the center of the column matrix was added with 50 µL of preheated elution buffer.
9. Let the tubes stand for at least 5 minutes to ensure the matrix absorbs the elution buffer. For purified DNA, they were centrifuged at 10,000 rpm for 30 seconds.

### 2.6. Primer selection of the SNP of IL-1β (rs16944) using conventional polymerase chain reaction (PCR)

This study designed ARMS-PCR primers for the (rs16944) gene polymorphism using the NCBI—SNP database and Primer1 ARMS-PCR primers designed online. (Scientific Researchers Co., Ltd., Baghdad, Iraq), provided these primers, as seen in Table 1.

**Table 1:** The sequencing of primer.

Primer	Sequence (5’–3’)	Product size
Wild-type forward primer	GGTGCTGTTCTCTGCCTCA	188 bp
Mutant forward primer	GGTGCTGTTCTCTGCCTCG	
Common reverse primer	TGAGATTGGCTAGGGTAACA	

ARMS-PCR master mix was prepared using the GoTaq® G2 Green Master Mix kit, according to company instructions; after that, PCR master mix components were transferred into Exispin vortex and centrifuged at 3000 rpm for 3 minutes and then placed in a PCR Thermocycler (BioRad Laboratories, Hercules ). The PCR Thermocycler conditions are illustrated in Table 2.

**Table 2:** PCR thermocycler conditions.

PCR step	Temperature	Time	Repeat
Initial denaturation	95°C	5 min	1
Denaturation	95°C	30 s	35 cycle
Annealing	55°C	30 s	
Extension	72°C	30 s	
Final extension	72°C	5 min	1
Hold	4°C	Forever	–

PCR = polymerase chain reaction.

### 2.7. ARMS-PCR product analysis

The ARMS-PCR products were examined using Agarose gel electrophoresis, which involved sealing the ends of the tray and placing an appropriate comb on the tray. The agarose gel was prepared by dissolving it in a buffer solution and heating it in a water bath at 100°C for 15 minutes. It was then allowed to cool down to 50°C. Next, the Agarose gel solution was introduced to 3 µL of ethidium bromide stain. The agarose gel solution was poured into the tray, and the comb was fixed appropriately. Subsequently, the solution was allowed to harden for 15 minutes at room temperature, after which the comb was carefully removed from the tray. The gel tray was securely positioned in the electrophoresis chamber and filled with buffer. Ten microliters of PCR products were added to each comb well, and 3 µL of (100 bp Ladder) were added to the first well. The electric current was conducted at 100 V and an amperage of 80 amps for 1 hour. The ARMS-PCR products were seen using a UV transilluminator.^[30]^

### 2.8. Ethical considerations

The study was approved by the Research Ethical Committee of the College of Medicine, Al-Nahrain University, with approval number (UNCOMIRB20240422) and data (November 20, 2022). Written informed consent was obtained from all study participants, per the Helsinki Declaration and its later amendments.

### 2.9. Sample size

The G-power program was used to calculate the sample size, with 81% power, 0.05 alpha level, an effect size of 0.4, a *t*-test family, and the total sample was 160.^[31,32]^

### 2.10. Statistical analysis

This case-control report was analyzed statistically with (SPSS version 24.0, Armonk) and (GraphPad Prism version 10.3.0, San Diego) for Windows. All data followed a normal distribution, and one-way ANOVA was used. Binary logistic regression analysis was used to calculate the odds ratio and its 95% confidence interval when the outcome can be categorized into 2 binary levels. The *P*-value was considered significant if ≤.05.

The genotyping results were analyzed, and frequencies of alleles and genotypes were calculated. Hardy–Weinberg equilibrium calculator for 2 alleles using an online calculator (https://www.had2know.org/academics/hardy-weinberg-equilibrium-calculator-2-alleles.html) using the difference in distribution between the actual frequency of genotype compared to the expected frequency of genotype^[33]^; IL-1β gene (rs16944) in both groups adheres to Hardy–Weinberg equilibrium.

## 3. Result

One hundred sixty participants completed the study, and the mean age and body mass index (BMI) were significantly higher in the diabetic patients; there was no significant difference in sex distribution between the 2 groups, as shown in Table 3.

**Table 3:** Demographic data.

Parameters	Control	T2DM	*P*-value
Number	74	86	–
Age (yr), mean ± SD	44.6 ± 10.4	51.8 ± 10.7	<.001
BMI (kg/m^2^), mean ± SD	27.2 ± 4.3	29.1 ± 4.8	.011
Sex			
Female	45 (60.8%)	55 (64.0%)	.682
Male	29 (39.2%)	31 (36.0%)	
Duration of DM	–	3.0 (0.41–5.25)	–

BMI = body mass index, T2DM = type 2 diabetes mellitus.

**Figure 1.**
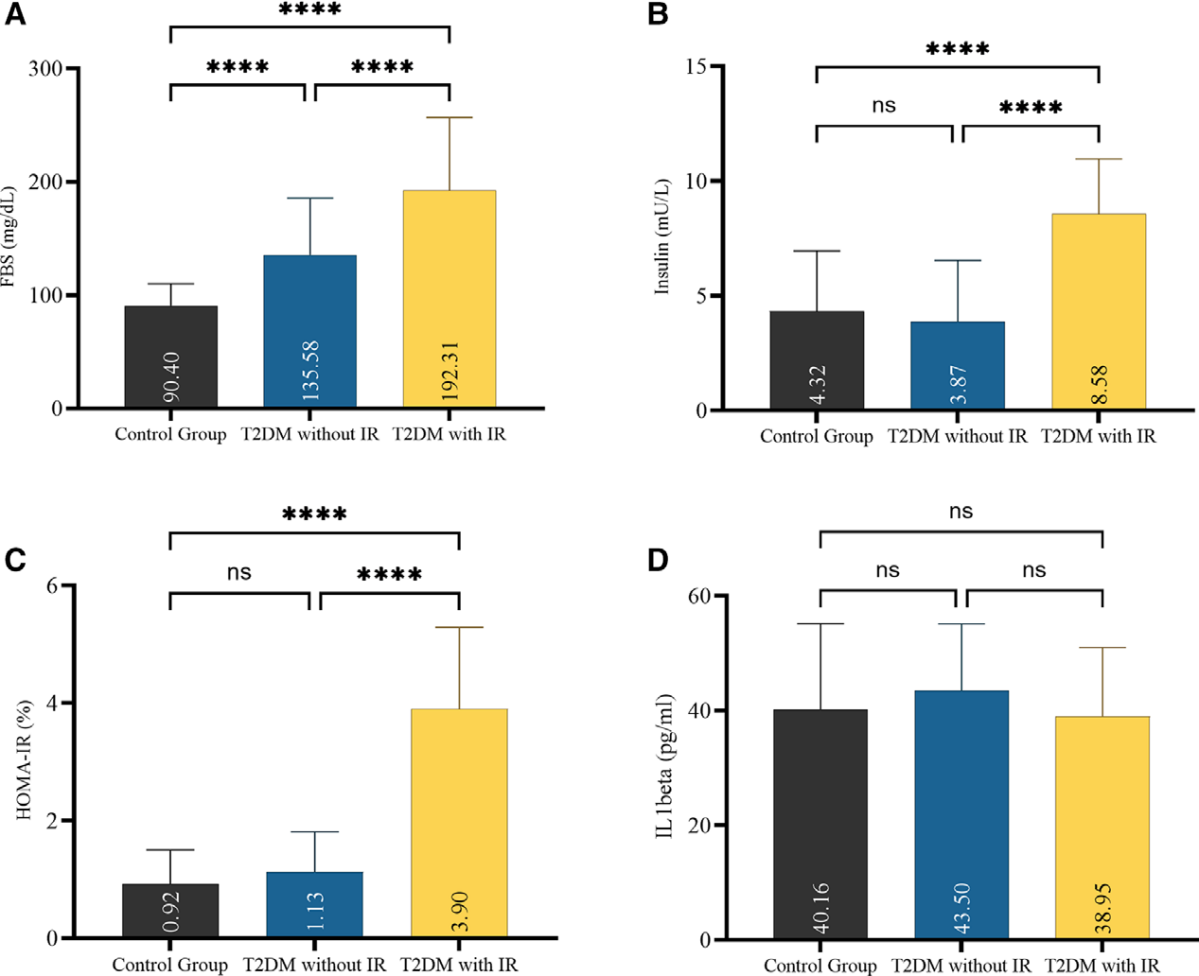
(A) FBS levels, (B) insulin levels, (C) HOMA-IR, and (D) IL-1β levels according to the studied groups. FBS = fasting blood sugar, HOMA-IR = homeostatic model assessment for insulin resistance, IL = interleukin.

The FBS level has been shown to differ significantly among different groups. Both insulin and homeostatic model assessment for insulin resistance (HOMA-IR) showed significantly elevated levels in patients with IR and T2DM compared to control and T2DM without IR. Lastly, IL-1β did not differ between study groups, as shown in Figure 1.

### 3.1. Variation of IL-1β (rs16944)

**Figure 2.**
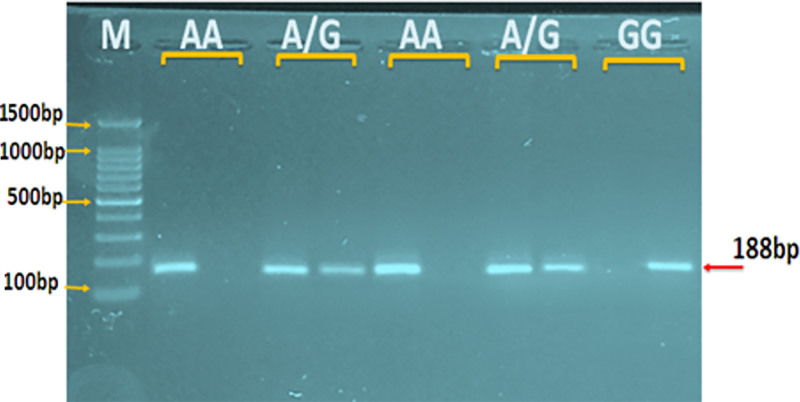
Agarose gel electrophoresis image that showed the ARMS-PCR product analysis of rs16944 (A/G) gene polymorphism. Where M: marker (1500–100 bp). The presence of A or G alleles was observed at 281 bp product size. The (AA) wild-type homozygote was shown in an allele only, the (GG) mutant-type homozygote was shown in the G allele only, whereas the (A/G) heterozygote was shown in both A and G alleles.

The obtained DNA was analyzed using ARMS-PCR, a technique that utilizes specialized primers designed to target a particular area of the DNA. The gel electrophoresis technique was used to identify the genetic variants in the examined SNPs. IL-1β has 3 genotypes: heterozygous (AG), homozygous (GG), and wild (AA), as shown in Figure 2.

### 3.2. Distribution of the association of IL-1β gene polymorphism (rs16944) according to the groups

Interleukin-1β SNP (rs16944, A > G) variations in the control group showed 32 (43%) of AA, 27 (36%) of AG, and 15 (20%) of GG, while T2DM patients showed 30 (35%) of AA, 34 (40%) of AG and 22 (26%) of GG variation. The logistic regression analysis showed a non-significant association between each variation and T2DM. Also, the allelic distribution of A and G did not show significant differences between the control and T2DM groups, as shown in Table 4.

**Table 4:** Assessment if IL-1β gene polymorphism (rs16944).

IL-1β variations	Control	T2DM	OR	95% CI	*P*-value*
GG	15 (20.3%)	22 (25.6%)	1.564	0.686–3.566	.287
AG	27 (36.5%)	34 (39.5%)	1.343	0.661–2.731	.415
AA	32 (43.2%)	30 (34.9%)	1.0	–	–
IL-1β alleles					
G	57 (38.51%)	78 (45.35%)	1.325	0.8544–2.073	.2170
A	91 (61.49%)	94 (54.65%)	1.0	–	–

CI = confidence interval, OR = odd ratio, T2DM = type 2 diabetic mellitus.

* Binary logistic regression.

### 3.3. Association of (rs16944) with control and IR DM2

The genome variation in patients with IR and T2DM was 8 (33%) AA, 6 (25%) AG, and 10 (42%) GG. The logistic regression test showed a non-significant association between each variation and patients with IR and T2DM. Also, the allelic distribution of A and G alleles showed non-significant differences between control and T2DM with IR patients, as shown in Table 5.

**Table 5:** Assessment of IL-1β gene polymorphism (rs16944) between control and T2DM with IR patients.

IL-1β variations	Control	T2DM with IR	OR	95% CI	*P*-value*
GG	15 (20.3%)	10 (41.7%)	2.667	0.876–8.122	.084
AG	27 (36.5%)	6 (25.0%)	0.889	0.274–2.881	.844
AA	32 (43.2%)	8 (33.3%)	1.0	–	–
IL-1β alleles					
G	57 (38.51%)	26 (54.17%)	1.887	0.962–3.562	.057
A	91 (61.49%)	22 (45.83%)	1.0	–	–

CI = confidence interval, IR = insulin resistance, OR = odd ratio, T2DM = type 2 diabetic mellitus.

* Binary logistic regression.

## 4. Discussion

Several clinical investigations have established a significant correlation between the onset of T2DM and several proinflammatory markers, including IL-6, IL-1β, and TNF-α.^[34–36]^ Chronic exposure to high levels of glucose and lipids usually elicits countless pathways responsible for impaired insulin secretion from the β-cells, leading to a decrease in glucose utilization peripherally.^[34]^

The current study showed that FBS levels in T2DM with IR patients are higher than those of T2DM without IR patients, with a significant value, and both are significantly different compared to the control group and between T2DM with or without IR groups. A study conducted by Misaki et al in Japanese patients showed that circulating levels of IL-1β were positively correlated with FBS levels.^[37]^ Fascinatingly, the mutant genotype of IL-1β SNP (rs16944) was also documented to be associated with an increase in the risk of glucose homeostasis in T2DM patients.^[38]^ Another study showed a significant relationship between IL-1β rs16944 and elevation of FBS; this study does not agree with the current results.^[27]^

The results of the current study regarding insulin levels were found to be significantly elevated in the T2DM with IR group, and non-significant in the non-IR group and the control group. The findings of our study align with the collective research undertaken by other research teams, which examined the insulin levels in each of the groups under study.^[39–41]^ Regarding insulin levels in association with IL-1β in the current study, the highest insulin level was recorded in the T2DM with IR group, and the lowest in the T2DM without IR group. It has been proven that IL-1β plays a role in reducing insulin production by impairing β-cell function. On the other hand, chronic stimulation of insulin secretion may exhaust β-cells.^[42]^ Banerjee and Saxena reported that IL-1β, a proinflammatory factor, inhibits β-cell function and promotes β-cell apoptosis.^[43]^ Vesa et al reported that dropping insulin secretion in newly diagnosed T2DM patients may be due to the elevation of IL-1β level, which affects beta cell function.^[44]^

Homeostatic model assessment for IR is the index used to identify IR or insulin sensitivity in T2DM, derived by evaluating the relationship between FBS and Fasting insulin.^[45,46]^ The current study demonstrated an increase in HOMA-IR in the IR group compared to the control group; this notable increase in IR results from elevated glucose and insulin levels caused by a malfunction in the insulin signaling system. This fact is substantiated by a compilation of studies bolstered by the findings of our research.^[47–50]^ Furthermore, the relationship between BMI and HOMA-IR is supported by many studies that report overweight conditions may be considered a risk factor for the development of IR.^[48,51,52]^

In the current study, levels of IL-1β showed non-significant differences among the studied groups; the non-significant differences in IL-1β levels among studied groups may have occurred due to less effect of IL-1β on each insulin and FBS levels, as well as glucose uptake. On the other hand, our results regarding IL-1β levels may be due to the small sample size. A study by Welsh et al supports the current results that showed no difference between patients and controls in IL-1β protein levels and gene expression.^[53]^ Another study, consistent with our result, conducted by Linhartova, showed no significant differences between control and patient groups in IL-1β levels.^[54]^ Conversely, Atieh et al reported significant differences in IL-1β levels between control and patient groups.^[55]^ Böni-Schnetzler reported that the concentration of IL-1β in patients with T2DM is so low that the inflammation by IL-1β cannot be evaluated in T2DM patients.^[23]^ Another study conducted by Spranger et al reported that IL-1β insufficient levels lead to the development of T2DM and that combining IL-1β with another proinflammatory cytokine may enhance T2DM development.^[56]^

Regarding the genetic investigation of IL-1β (rs16944), it showed 3 variations, which are heterozygous (AG), homozygous (GG), and wild type (AA); showed a non-significant association between each variation and the incidence of T2DM with IR. This test indicated that the largest portion was detected in the GG variant, followed by the AA and AG variants. Also, the allelic distribution of A and G alleles showed non-significant differences among the studied groups, possibly due to the small sample size. Contrary to our findings, another study done in Egypt to study IL-1β (rs16944) reported a strong relationship between IL-1β (rs16944) and T2DM in 30 healthy control and 50 patients with T2DM individuals and given a strong association of mutant genotype (CC) in T2DM was much higher in T2DM patients than in healthy controls.^[57]^ However, previous studies in the Indian population showed that the association of IL-1β CT with T2DM and TT genotype is highly significant in patient groups rather than the control groups.^[58]^ While another study from the Gujarat region in India observed that IL-1β (rs16944) was not associated with T2DM, although elevated IL-1β transcript levels in patients might confer additional risk toward the disorder.^[59]^

The current result is supported by a study conducted by Ismail et al in the Malaysian population that showed no significant association between C-511T and variant IL-1β in T2DM patients and control groups.^[60]^ However, Welsh et al showed that neither high glucose in vitro nor the diabetic state in vivo led to IL-1β production in human islets. Collectively, these results suggest that local secretion of IL-1β is not necessarily associated with the glycemic status in diabetes.^[47]^ Böni-Schnetzler et al reported that the promoter region of IL-1β (rs16944) polymorphism may modify the expression of IL-1β; also, there is no difference between the C and T allele distribution. Furthermore, it reported that IL-1β levels can be associated with potent inflammatory responses in such patients.^[23]^

Although the study stratified patients by the rs16944 genotype (AA, AG, and GG), the data did not reveal any significant differences in circulating IL-1β protein levels between these groups. Such a finding suggests that the promoter SNP (rs16944) may not be the sole or dominant regulatory element controlling IL-1β expression in peripheral blood. It is conceivable that while this SNP has functional potential in altering gene transcription rates in some contexts, its effect might be overshadowed by other layers of regulation (e.g., epigenetic modifications, post-transcriptional regulation via microRNAs, or even post-translational processing). In other words, the genotype might not translate into the expected differences in protein levels due to compensatory mechanisms operating within the immune regulatory network.^[27]^

IL-1β is known to have autocrine and paracrine effects, particularly within pancreatic islets, where its local expression may be more critical than its serum levels. It is possible that the rs16944 variation may influence IL-1β expression locally – in the pancreatic tissue or other specific microenvironments – without markedly altering circulating levels. The systemic measurement of IL-1β might thus not capture genotype-dependent differences that occur at the tissue level.^[61,62]^

The inflammatory response in T2DM involves a complex network of cytokines and pathways. Even if the rs16944 SNP modulates IL-1β transcription to some extent, the overall inflammatory milieu (which includes IL-6, TNF-α, and other mediators) can mask or override the isolated effects of this single polymorphism. Feedback loops and the interplay among various cytokines likely contribute to the observed non-significant differences.^[24,63]^

Finally, we conclude that the proinflammatory factor IL-1β, at both genetic and protein levels, did not reveal a relation with the development of such a disease. Indeed, our result may need to be supported by studying other factors that may impact the regulation of the hyperglycemic condition associated with this disease.

### 4.1. Study limitations

First, there were significant differences in BMI between the control and T2DM groups. Obesity is well known to drive low-grade chronic inflammation and can independently elevate IL-1β production. This difference may confound the relationship between genotype and cytokine levels, as the inflammatory signal from adipose tissue could mask any genotype-dependent modulation. Second, the T2DM patients were, on average, older than the control subjects. Age-related changes in immune function, variations in dietary patterns, physical activity levels, and other lifestyle factors can impact cytokine production. These factors may be unevenly distributed between the groups and confound any direct genotype–phenotype associations. Third, many individuals with T2DM are on various medications (anti-diabetic drugs, statins, anti-hypertensives, etc), which could influence inflammatory markers. In addition, although the study excluded patients with acute inflammatory conditions, chronic conditions (such as subclinical infections or other comorbidities) might influence IL-1β levels without being accounted for. And finally, while the study provides useful insights into IL-1β protein levels and the rs16944 polymorphism, its limited sample size and the further stratification by IR mean that the non-significant findings could stem from inadequate power rather than a true absence of association. Future studies with larger cohorts or meta-analytic approaches would be beneficial for confirming these findings and better elucidating any potential genetic links.

## 5. Conclusion

In this case-control study of an Iraqi cohort, we found no significant association between IL-1β protein levels or the rs16944 polymorphism and measures of blood sugar metabolism or IR in T2DM. Although metabolic parameters, such as FBS, insulin, and HOMA-IR, differed significantly between the diabetic and control groups, IL-1β levels and its genetic variants (AA, AG, and GG) remained comparable. This suggests that, despite its established role in inflammation and β-cell dysfunction, IL-1β may not directly influence the hyperglycemic state or IR in this population. Given the limited sample size, further studies with larger cohorts and an expanded set of inflammatory markers are warranted to clarify the complex interplay between genetic factors, cytokine regulation, and the pathogenesis of diabetes.

## Author contributions

**Conceptualization:** Haider Mohammad Khdaer, Muhammed A. H. Aldabagh, Zeyad T. Abdulrazzaq, Galal A. Altai, Ali Abdullah Issa, Majid S. Jabir, Mazin A. A. Najim, Sabrean F. Jawad, Hayder Adnan Fawzi, Samer Tareq Jasim, Ayman A. Swelum.

**Data curation:** Haider Mohammad Khdaer, Muhammed A. H. Aldabagh, Sabrean F. Jawad, Hayder Adnan Fawzi.

**Formal analysis:** Hayder Adnan Fawzi, Samer Tareq Jasim.

**Funding acquisition:** Ayman A. Swelum.

**Investigation:** Muhammed A. H. Aldabagh, Galal A. Altai, Mazin A. A. Najm, Thikra F. Hasan, Hayder Adnan Fawzi.

**Methodology:** Haider Mohammad Khdaer, Muhammed A. H. Aldabagh, Zeyad T. Abdulrazzaq, Galal A. Altai, Sabrean F. Jawad, Samer Tareq Jasim.

**Project administration:** Haider Mohammad Khdaer, Zeyad T. Abdulrazzaq.

**Resources:** Haider Mohammad Khdaer, Muhammed A. H. Aldabagh, Galal A. Altai, Ali Abdullah Issa, Sabrean F. Jawad, Hayder Adnan Fawzi, Samer Tareq Jasim.

**Software:** Haider Mohammad Khdaer, Muhammed A. H. Aldabagh, Zeyad T. Abdulrazzaq, Majid S. Jabir, Hayder Adnan Fawzi.

**Supervision:** Majid S. Jabir.

**Validation:** Haider Mohammad Khdaer, Ali Abdullah Issa, Mazin A. A. Najm, Thikra F. Hasan, Ayman A. Swelum.

**Visualization:** Haider Mohammad Khdaer, Mazin A. A. Najm, Thikra F. Hasan, Ayman A. Swelum.

**Writing – original draft:** Haider Mohammad Khdaer, Muhammed A. H. Aldabagh, Zeyad T. Abdulrazzaq, Galal A. Altai, Ali Abdullah Issa, Majid S. Jabir, Mazin A. A. Najm, Thikra F. Hasan, Sabrean F. Jawad, Hayder Adnan Fawzi, Samer Tareq Jasim, Ayman A. Swelum.

**Writing – review & editing:** Haider Mohammad Khdaer, Muhammed A. H. Aldabagh, Zeyad T. Abdulrazzaq, Galal A. Altai, Ali Abdullah Issa, Majid S. Jabir, Mazin A. A. Najm, Thikra F. Hasan, Sabrean F. Jawad, Hayder Adnan Fawzi, Samer Tareq Jasim, Ayman A. Swelum.
